# Concatenated alignments and the case of the disappearing tree

**DOI:** 10.1186/s12862-014-0266-0

**Published:** 2014-12-30

**Authors:** Thorsten Thiergart, Giddy Landan, William F Martin

**Affiliations:** Institute of Molecular Evolution, Heinrich-Heine-Universität Düsseldorf, Düsseldorf, Germany; Genomic Microbiology Group, Institute of Microbiology, Christian-Albrechts-Universität Kiel, Kiel, Germany

**Keywords:** Phylogeny, Concatenation, Conflicting signals, Bootstrapping

## Abstract

**Background:**

Analyzed individually, gene trees for a given taxon set tend to harbour incongruent or conflicting signals. One popular approach to deal with this circumstance is to use concatenated data. But especially in prokaryotes, where lateral gene transfer (LGT) is a natural mechanism of generating genetic diversity, there are open questions as to whether concatenation amplifies or averages phylogenetic signals residing in individual genes. Here we investigate concatenations of prokaryotic and eukaryotic datasets to investigate possible sources of incongruence in phylogenetic trees and to examine the level of overlap between individual and concatenated alignments.

**Results:**

We analyzed prokaryotic datasets comprising 248 invidual gene trees from 315 genomes at three taxonomic depths spanning gammaproteobacteria, proteobacteria, and prokaryotes (bacteria plus archaea), and eukaryotic datasets comprising 279 invidual gene trees from 85 genomes at two taxonomic depths: across plants-animals-fungi and within fungi. Consistent with previous findings, the branches in trees made from concatenated alignments are, in general, not supported by any of their underlying individual gene trees, even though the concatenation trees tend to possess high bootstrap proportions values. For the prokaryote data, this observation is independent of phylogenetic depth and sequence conservation. The eukaryotic data show much better agreement between concatenation and single gene trees. LGT frequencies in trees were estimated using established methods. Sequence length in individual alignments, but not sequence divergence, was found to correlate with the generation of branches that correspond to the concatenated tree.

**Conclusions:**

The weak correspondence of concatenation trees with single gene trees gives rise to the question where the phylogenetic signal in concatenated trees is coming from. The eukaryote data reveals a better correspondence between individual and concatenation trees than the prokaryote data. The question of whether the lack of correspondence between individual genes and the concatenation tree in the prokaryotic data is due to LGT or phylogenetic artefacts remains unanswered. If LGT is the cause of incongruence between concatenation and individual trees, we would have expected to see greater degrees of incongruence for more divergent prokaryotic data sets, which was not observed, although estimated rates of LGT suggest that LGT is responsible for at least some of the observed incongruence.

**Electronic supplementary material:**

The online version of this article (doi:10.1186/s12862-014-0266-0) contains supplementary material, which is available to authorized users.

## Background

Constructing trees out of concatenated alignments is now common practice in phylogenetics [1,2]. A problem encountered in some of the earlier concatenation studies is that the concatenation tree is fully supported via bootstrapping at many or all branches but trees for the individual genes do not support the concatenation result, or conflict with it [3,4]. In investigations of prokaryotic gene trees, the topological differences between individual trees underlying a concatenation are usually ascribed to lateral gene transfer (LGT) [5], which is not unreasonable, because prokaryotes really do undergo LGT frequently and have several biochemically and genetically well-characterized mechanisms to spread their genes within and across taxonomic boundaries: conjugation, transformation, transduction and gene transfer agents [6].

However there are other potential sources of phylogenetic conflict between gene trees and concatenated alignment trees. One of them is uncertain orthology or hidden praralogy. For example, Rinke et al. [7] examined a tree of concatenated alignments comprising newly characterized archaeal lineages, the concatenated result recovered the familiar three domains tree, with eukaryotes branching as sisters to archaebacteria. Williams and Embley [8] reinspected that data and found that the sequence collection procedure used by Rinke et al. [7] had included several nuclear genes of mitochondrial and plastid origin among the eukaryotic sequences; when those were removed and replaced by eukaryotic nuclear genes that had not been acquired from mitochondria or plastids, the two-domain tree was obtained [8], in which eukaryotes branch within the archaea [9]. Another source of conflict is phylogenetic error due to unknown factors that are often subsumed into the term model misspecification. For sequences from 10 plastid genomes, where neither paralogy nor orthologous replacement of sequences via LGT are known to occur, the species tree was fully resolved by the concatenation of 42 protein coding plastid gene families, but only 11 of the 42 gene trees recovered the concatenated topology, the remainder supported different trees [4]. The reason for the differing results are best explained by the circumstance that different proteins undergo amino acid substitution in different ways over evolutionary time, and according to different processes, models for which can be approximated mathematically [10,11].

One of the more controversial applications of alignment concatenation concerns its use to construct phylogenies for prokaryotes. At the center of the debate is the question whether there is a meaningful phylogeny of prokaryotes or not [12,13] and if so, does it extend back to the depths of evolutionary time [14], or does a tree only exist for the tips of prokaryotic trees [15]. In genomes, there exists a set of about 33 genes that are universally conserved among prokaryotes and that can readily be identified using standard ("manual") sequence comparison procedures [16,17]. The existence of that universal set has been confirmed using semi-automated procedures [18]. Concatenation of those alignments produces a tree [16-18], but individually, the proteins in question do not tend to support any particular branching order, especially for the deeper branches or prokaryote phylogeny [19,20].

Why do concatenation trees that are strongly supported, in terms of bootstrap proportions, fail to be supported by the individual gene trees constructed from the same underlying data? We reasoned that if LGT is the cause of conflict between individual gene trees, then its effect should be greater in prokaryotic than in eukaryotic data sets of similar sequence divergence, because LGT is far more prevalent among prokaryotes than it is among eukaryotes [21]. If model misspecification is the cause, then prokaryotic and eukaryotic data sets of similar sequence divergence should show similar levels of conflict. In prokaryote genomes, analyses of more closely related prokaryotic sequences should uncover greater congruence than for more distantly related prokaryotic sequences, because accurate phylogenetic inference becomes more problematic as sequence divergence increases [9] and because both LGT and sequence divergence accumulate over time [22]. In an effort to discriminate these possible causes, we undertook investigations of real data analyzed as individual and concatenated alignments.

## Methods

### Data

Proteome datasets were downloaded from RefSeq database [23]. These were: 1606 prokaryotic proteomes (v03.2012), 81 fungal proteomes (v03.2012), 86 animal proteomes (v03.2013) and 22 plant proteomes (v03.2013).

### Gene families

Prokaryotic gene families were retrieved from the clusters of orthologous groups database (COGs, [24]). To avoid bias of the sampling and ensure an even taxonomic representation of the major taxonomic phyla of both prokaryotic domains, 50 archaeabacterial and 50 eubacterial genomes were chosen for further analysis. We avoided highly reduced genomes in our sample and were thus able to identify 48 genes that were present in a sample of 100 prokaryotic genomes containing 50 bacteria and 50 archaea (Additional file 1). Homologues gene sequences of these 48 gene families were collected for two additional datasets, one containing 100 proteobacteria (Additional file 2) and one containing 100 gammaproteobacteria (Additional file 3). Additionally, a search was performed within all gammaproteobacteria species, yielding a dataset comprising 100 gammaproteobacteria species (Additional file 4) and 200 universal gene families. For comparions between eukaryotic datasets and gammaproteobacteria data, this dataset was pruned to 50 taxa (Additional file 4).

Two datasets were generated for the eukaryotic analysis: one comprising only fungal species, and one containing plant, fungal and animal sequences. Universal protein families were reconstructed by an initial search for similar proteins with BLAST [25]. BLAST hits above 35% identity, an e-value ≤10^−10^ and an alignment length ≥ 75 were retained. Sequence pairs with ≥30% global identity using the needle algorithm (EMBOSS-package, [26]) were used as input for clustering with MCL [27]. Protein families were then sorted according to their universality. The first 200 families were chosen for the fungal set (50 species, Additional file 5), 79 universal protein families were retrieved from the mixed eukaryote set (50 species, Additional file 6). A taxonomic flittering procedure was applied on both datasets to reduce oversampling. To filter for possible paralogous sequences in all datasets, the subset of all possible paralogs/orthologs that have the smallest reciprocal distance and that included all species having multiple copies was chosen. Clusters in which the subset did not include all species were not considered further.

### Alignments and phylogenetic methods

Sequences were aligned with MAFFT (multiple alignment using fast Fourier transformation, v6.832b) using the “G-INS-I” parameters [28]. Trees were constructed with RAxML v7.0.4 [29]. The substitution rate per site was estimated from a gamma distribution with four discrete rate categories and the WAG substitution matrix [30]. The proportion of invariable sites was estimated from the data. Concatenated alignment trees were generated from the original alignments for the different datasets. Phylogenetic trees from prokaryotic datasets were rooted i) between archaea and bacteria, ii) using epsilonbacteria as the outgroup for the proteobacterial dataset or iii) using *Francisella* sp. as outgroup for the gammaproteobacteria. Phylogenetic trees from the eukaryotic datasets were rooted between plants and fungi/animals or between ascomycetes and basidiomycetes in the case of the fungi dataset. Full species names and additional taxonomic information are given in Additional files 1, 2, 3, 4 and 5. To test for potential lateral gene transfer in our datasets we used PRUNIER [31] and RANGER-DTL [32]. All 200 trees from the gammaproteobacteria data and the fungi data including 50 species were tested, respectively. Both programms require a reference tree, to which the single gene trees are compared, therefore all the 200 alignments from each dataset were concatenated to produce a reference tree. PRUNIER calculates several possible LGT scenarios, we selected the one that showed the smallest amount of LGT.

### Simulations

Simulated alignments were created using a modified DAWG [33] version that is able to simulate evolution of amino acid sequences. The input tree was obtained from the weighted concatenated alignment of the γ-proteobacteria dataset, consisting of the 48 conserved genes. Datasets with an alignment length of 200 and 1000 positions were simulated, using the following DAWG parameters: Tree Scale = 1, SubsModel = WAG, IndelModel = zipf, Indel Param 1.6, 100, Indel Rate 0.0011. This specific indel rate was used to match the one obtained for the alignments that originated the input tree.

### Statistical analysis

All informative splits that were present in a given set of gene family trees were referred as the split pool. Pairs of splits were classified as compatible, when they can occur simultaneously in a binary tree, and classified as incompatible otherwise [34]. For each node in the concatenation trees, the amount of identical nodes within gene family trees were counted. This value is termed the node score. The presence of a node in two trees implies that the three splits that are connected at the node are present in both trees. The topological distance from a node to the tip of a tree was calculated as the average number of branches separating a node from its descendant leafs. All statistical tests were performed using Matlab. Correlation measurements were done using the Pearson’s linear correlation coefficient. To test if the difference between node score values for different datasets is significant, we used the MATLAB *multcompare* function (based on a one-way analysis of variance, alpha 0.05). To find subsets of similar trees, we used the number of different splits between two trees as a distance measurement and pass this data to the *linkage*/*cluster* functions in MATLAB to receive hierarchical ordered clusters.

## Results

### The disappearing tree phenomenon

**Figure 1:**
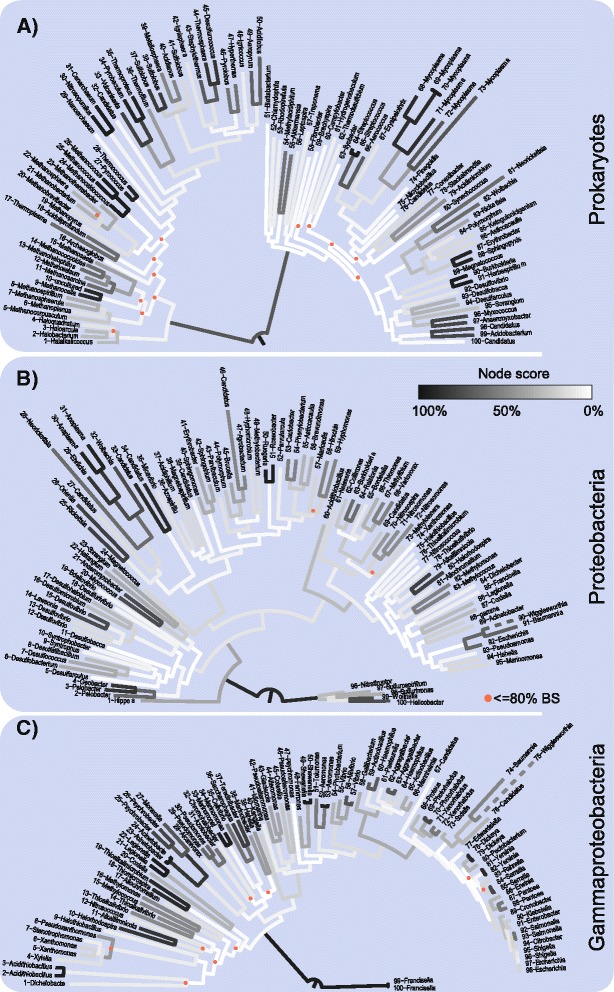
**Single gene tree support projected on three concatenated prokaruyotic trees of different taxonomic depth levels.** All trees based on the concatenation of 48 universal genes. Nodes in concatenated trees were compared with nodes present in the underlying single gene trees. Each node and their outgoing branches were colored according to presence of this node within single gene trees, from 0 to all 48 single gene trees. The trees include **A)** a prokaryotic dataset including 100 archaebacteria and eubacteria, **B)** 100 proteobacteria, **C)** 100 gammaproteobacteria species. Exact species names are given in Additional files 1, 2 and 3.

Concordance between the branches in individual gene trees and their concatenated incarnation is weak, as suggested by earlier studies [5,35]. For the present data, this is shown in Figure 1, using a dataset of 48 genes present in three samples of 100 prokaryotic genomes spanning three phylogenetic depths: 50 archeabacteria and 50 eubacteria (Figure 1A), 100 proteobacteria (Figure 1B), and 100 gammaproteobacteria (Figure 1C). In each 100 genome, 48 gene sample, the frequency of branches in 48 individual gene trees were compared to the set of branches in the concatenation tree. For each internal node within the concatenation tree, the node score was specified as the number of times that the corresponding node was observed among the 48 individual gene trees.

For the most divergent data set (Figure 1A), deeper internal nodes of the concatenated tree have almost no congruence with the nodes in single gene trees, except the branch separating archaebacteria and eubacteria. At the tips of the tree, much greater congruence between the individual genes and the concatenation tree is observed. Surprisingly, the same "tree of tips" [15] or "disappearing tree" [35] phenomenon was observed for the proteobacterial sample (Figure 1B) and for the gammaproteobacterial sample (Figure 1C). For all three samples of phylogenetic/taxonomic depth, congruence between the deeper internal branches and branches recovered in individual trees disappears, yet the bootstrap proportions (BP) for virtually all branches in the concatenation trees were very high: for all three concatenation trees combined, only 15 internal branches had a BP *below* 80 (nodes marked with a red dot in Figure 1) and the average BP was 90, 99, and 96 for Figure 1a-c respectively.

For the deep prokaryote sample, there are mainly two areas of low BPs within the tree: one within the euryarchaeota, and one spanning firmicutes, actinobacteria and tenericutes. The corresponding node support values relative to individual trees are low as well. But it is clearly visible that the rest of the internal branches have low node scores (congruence among individual trees) and high bootstrap support (site pattern sampling in the concatenation tree). The total number of splits present within the 48 single gene trees (split pool), is a simple measure to reflect the observed incongruence within the concatenation tree. On the range between total congruence with a split pool of 97 splits and total incongruence with 4,656 possible splits, 1,830 different splits were observed for the set of 48 trees summarized in Figure 1A. For trees in Figure 1B and C, 1,905 and 1,804 splits were observed, respectively. In other words, each internal branch of the species tree generates more than 18 conflicting splits on average. Especially for deeper phylogenetic relationships, the topology in the concatenation tree is not present in any of the family gene trees, despite the corresponding branches of the concatenation tree showing high BP values.

### Influence of LGT – Comparing eukaryotic and prokaryotic data

The main effect of LGT on prokaryote genome evolution is to alter the number and kinds of genes that are found in a prokaryotic genome, not to promote orthologous replacement [36]. But there is also evidence that some of the core genes in prokaryotes might be replaced during evolution [37-39]. Thus, if LGT is the main reason why the present set of prokaryotic "core" genes analyzed individually tend to obtain different phylogenetic results, then this tendency should be more pronounced in prokaryotes than in eukaryotes. This is because eukaryotes counteract Muller's ratchet using meiosis and sex (process that generate reciprocal recombination), while prokaryotes rely on mechanisms of LGT — transformation, conjugation and transduction — processes that spread genes unidirectionally from donors to recipients. In order to see whether the congruence between concatenation trees and individual phylogenies is greater in prokaryotes or eukaryotes, we compared two additional datasets: one comprising of 50 fungal genomes, and one comprising of 50 eukaryotes, spanning plants, animals and fungi (PAF). Both datasets were composed of 50 genes with comparable length and different average pairwise identities (61% in fungi, 49% in the mixed set).

**Figure 2:**
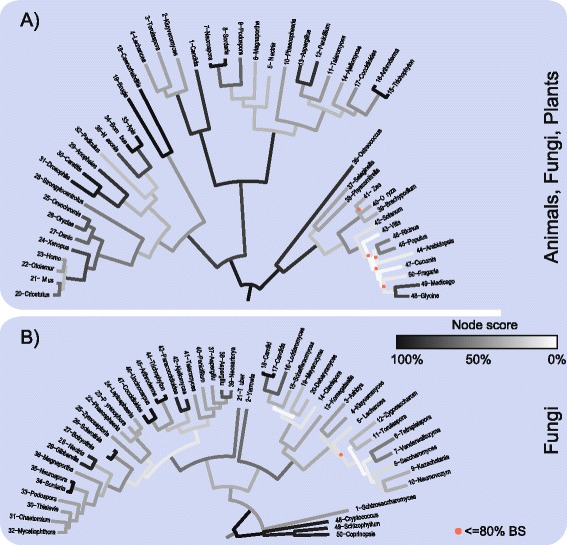
**Single gene tree support projected on two concatenated eukaryotic trees of different taxonomic depth levels.** All trees based on the concatenation of 50 universal genes, respectively. Nodes in concatenated trees were compared with nodes present in the underlying single gene trees. Each node and their outgoing branches were colored according to presence of this node within single gene trees, from 0 to all 48 single gene trees. The trees include **A)** 50 fungi, plant and animal species, **B)** 50 fungi. Exact species names are given in Additional files 6 and 5.

The results, summarised in Figure 2A and B, show that both eukaryotic concatenation trees tend to have weaker node scores in the deeper branches than at the tips, like the prokaryote concatenation trees, but the overall agreement between concatenation trees and individual gene trees is far better for the eukaryotic data than for the prokaryotic data. As in the prokaryotic example, the eukaryotic concatenation trees show high BPs, averaging 96 and 97, respectively. The PAF tree shows a clear correspondence between low bootstrap support and low node score in the clade spanning the higher plants. But, as in the case of the prokaryotic trees, sampling at increasing phylogenetic depth does not reduce the congruence between individual gene trees and concatenated trees, as the average node score, 25% ± 14, for the fungal data set (Figure 2B) is slightly higher than the value for the plant-animal-fungi dataset, 19% ± 11 (Figure 2A) (*P* = 0.026). Out of possible 2,350 splits we observed 350 different splits within the PAF dataset and 390 splits within the fungi dataset.

### Factors affecting node scores

**Figure 3:**
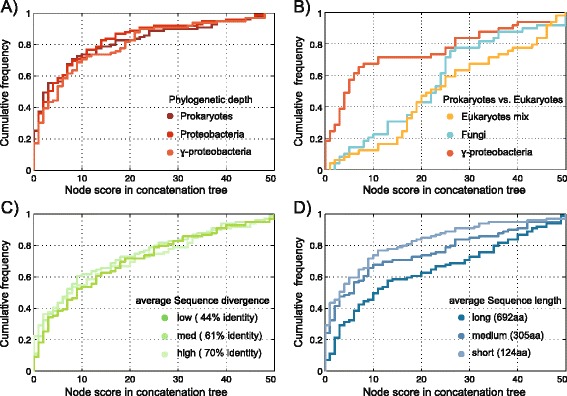
**Parameters influencing node score in concatenated trees and single gene trees.** Nodes from concatenated trees were plotted according to their support level compared to single gene trees. All datasets based on 50 single gene trees, except in **A)**, there are only 48 genes. **A)** Comparisons of the support level at different phylogenetic depths: prokaryotes, proteobacteria, gammaproteobacteria. **B)** Comparison of the support level for two eukaryotic and one prokaryotic dataset (all datasets, prokaryotic and eukaryotic, consists of 50 taxa), where the underlying single gene trees have similar average sequence length: Eukaryotes mixed: 438 ± 96 aa, Fungi: 441 ± 45 aa, gammaproteobacteria: 441 ± 57aa **C)** Comparison of the support level at different pairwise identity levels. Values are average percent identities in all pairwise sequence comparisons. **D)** Comparison of the node score for different average sequence lengths. Values are the average length of all protein sequences in each set.

We investigated different factors that might affect node scores, which are a proxy for the tendency of individual trees to recover branches found in the concatenated tree. For this, we plotted, for each node in the concatenation tree, the frequency with which it was recovered in different data samples in order of increasing frequency (abscissas in Figure 3).

First we looked at phylogenetic depth (Figure 3A) because distantly related groups have distantly related sequences, which are notoriously hard to align, and their phylogenetic analysis can be further hampered by substitution levels that can approach saturation or algorithmic biases such as long branch attraction. The prokaryotic datasets shown in Figure 1— prokaryotes, proteobacteria, gammaproteobacteria — encompass the same 48 genes, but because of their different phylogenetic depth, they span different levels of sequence divergence, the average pairwise identity being 32%, 48% and 67% respectively. Perhaps surprisingly, there is no significant difference (*P* = 0.67, *P* = 0.40, *P* = 0.70) between the node score distributions of the three samples (Figure 3A), despite the samples spanning a twofold decrease in average pairwise sequence identity. Thus, for these samples, phylogenetic depth is not a cause of low node scores.

In Figure 3B we plotted node score distributions for the eukaryotic data sets shown in Figure 2. The comparison of the plant-animal-fungi vs. the fungal samples also revealed no significant difference, such that, like the prokaryotic samples, increasing sequence divergence stemming from greater phylogenetic depth (52% average pairwise identity PAF vs. 58% fungi) had no detectable effect on node scores. To see if differences between prokaryote and eukaryote samples could be detected, we constructed a gammaproteobacterial sample with the same number of sequences and taxa (50) as the eukaryotic samples and consisting of genes with similar lengths (avg. 441 gammaproteobacteria, avg 438 PAF, avg. 441 fungi) and similar sequence conservation (avg. 58% for the gammaproteobacteria). Despite having very similar sequence atttributes as the eukaryotic samples, the node score distribution for the 50-genome gammaproteobacterial sample is strongly shifted towards lower values and is significantly different from that for the eukaryotes (*P =* 0.0007 *, P =* 1.96 × 10^−5^) (Figure 3B). This would be consistent with an effect of LGT in the gammaproteobacterial sample, but if so, it remains puzzling why we do not see a decrease in the prokaryotic node score with increasing phylogenetic depth (Figure 1, Figure 3A). Notably here the two eukaryote sets show no significant difference in their node score distribution (*P =* 0.2377).

Figure 3C shows the node score distributions for gammaproteobacterial gene samples of 50 genes each that were separated into three categories of sequence divergence (average pairwise sequence identity 44%, 61% and 70% respectively). No significant difference between the node score distributions was observed (*P =* 0.59*, P =* 0.86*, P =* 0.46). This suggests that sequence divergence at similar phylogenetic depth is not a factor affecting node score.

Another possible factor affecting generation of incongruent branches in individual and concatenated analyses is sequence length, or small site sample size. To check this, we assembled three more samples from the gammaproteobacterial data, each consisting of 50 genes for the same 100 species. The three samples consist of sequences with different average sequence length (124aa, 305aa, 692aa). The distributions of the node scores for the individual genes vs. the respective concatenation tree in two of the three samples are significantly different (*P =*3.8 x 10^−5^ , *P =*0.0172), with the longer sequences providing higher values than the shorter sequences (Figure 3D).

**Figure 4:**
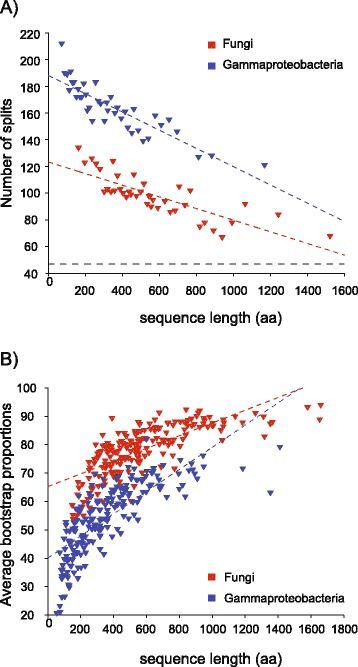
**Correlation between sequence length, tree incongruence and bootstrap proportions. A)** Number of different splits observed in bins of 5 trees is plotted against the average sequence length. **B)** Average bootstrap proportions within one tree plotted against the average sequence length. Both datasets, prokaryotic and eukaryotic, consists of 50 taxa. The polynomial regression is indicated as a colored dotted line. The black dotted line indicate the expected number of splits if all trees had identical topologies.

To investigate this effect further, we assembled fungal and proteobacterial datasets consisting of 200 genes each for 50 genomes and binned the individual alignments by their sequence length. In each of the 40 bins, we simply counted the number of different splits observed for the five trees in each bin. In the case of five identical topologies, we would observe 47 splits, in the case that no common branches were observed across all five trees in a bin, we would observe 235 splits. The numbers of splits observed in each bin are plotted against sequence length in Figure 4A. A very strong correlation is observed both for the gammaproteobacterial (*r* = −0.87, *P* = 2.2 × 10^−13^) and for the fungal bins (*r* = −0.8, *P* = 3.0 × 10^−10^). The corresponding analysis for alignment length, rather than sequence length, takes the influence of gaps into account, and very similar distributions to those obtained for sequence length were obtained (Additional file 7, gammaproteobacterial sample *r* = −0.78, *P* = 2.7 × 10^−9^, fungal sample *r* = −0.73, *P* = 5.6 × 10^−8^). Although these fungal and gammaproteobacterial samples have comparable sequence lengths and similar average pairwise identity distributions (fungi: 56% ± 5; gamma: 59% ± 6), the fungal data tends much more strongly to recover the same tree than the gammaproteobacterial sample does. This again might point to a greater role for LGT in the gammaproteobacterial genes than in the fungal genes sampled.

To estimate the presence of LGT in this data we used two established programms to search for potential LGT events, PRUNIER [31] and RANGER-DTL [32]. RANGER-DTL estimates the number of gene duplications, horizontal gene transfers and gene losses that are needed to reconcile a species and a gene tree. As a species tree we used a concatenation tree of all 200 genes for the fungi and gammaproteobacteria data. The mean number of estimated transfers per tree in the fungi data was 9, whereas it was 17.2 in the gammaproteobacteria data. PRUNIER uses a more conservative approach to estimate lateral transfer events, since it also includes a bootstrap cutoff. Using a cutoff of 70% yielded on average 2.3 transfers per tree within the fungi data and on average 3.3 transfers within the gammaproteobacteria data. Lowering the cutoff to 55% in the gammaproteobacteria data, since it has in average low BP values, yielded on average 5.7 transfers. The effect of LGT present in trees on incongruence is visible in Additional file 8. Again, length sorted bins were used to visualize the effect. This time gammaproteobacterial trees with low LGT rate (max. 1 event) and high LGT rate (at least 5) were compared. The low rate trees show higher congruency than the high rate trees. It might not explain all the difference between prokaryotic and eukaryotic data, but it shows that LGT has some influence as well.

Because bootstrapping provides information about the number of sites with similar distributions of site patterns needed to obtain the same tree in every pseudosample [40], it is perhaps not surprising that the average BP for each tree in the 200-gene gammaproteobacterial and fungal samples is strongly correlated with sequence length (Figure 4c). In the case of the gammaproteobacteria data (200 trees, all having the same 50 species), there is a strong positive correlation between bootstrap support in a gene family tree and sequence length (Figure 3B, *r* = 0.76, *P* = 5.72 × 10^−40^). A similar strong positive correlation between BP and sequence length is observed in the fungi dataset (Figure 3B, *r* = 0.73, *P* = 1.7 × 10^−35^). Other parameters do not show this strong correlation with BPs, or show no correlation at all. The alignment length has a slightly lower correlation with BP, than sequence length (gammaproteobacteria: *r* = 0.69, *P* = 2.23 × 10^−30^, fungi: *r* = 0.68, *P* = 1.06 × 10^−28^). The pairwise identity of genes within one gene family tree appears to correlate with BP (gammaproteobacteria: *r* = −0.31, *P* = 4.81 × 10^−6^, fun: *r* = −0.44, *P* = 3.5 10^−11^), but much less strongly than sequence length. Moreover, sequence length and pairwise identity are themselves only weakly or not correlated (fungi: *r* = −0.16, *P* = 0.017, gammaproteobacteria: *r* = −0.03, *P* = 0.598).

### Simulations to investigate the influence of sequence length

To see if the sequence length effect is repeatable with perfect alignments, we simulated alignments along a known evolutionary history. As an input for these alignments we used the concatenation tree made of the 48 conserved genes from 100 gammaproteobacteria species (Figure 1). Two datasets consisting of 50 alignments were generated, one with an initial alignment length of 1,000 positions, one with 200 positions. The dataset based on 1,000-position long alignments yield a nearly perfect distribution of splits. Nearly all of the 50 trees supporting the same splits, meaning all the trees are almost identical. The 200-position dataset trees have twice the amount of splits in their split pool than the longer ones (107 vs. 225 splits). To check whether the alignment process itself makes a difference, two additional datasets were made by recovering the sequences from the simulated alignment and aligning them using the same procedure as for the biological sequences. Again, no effect was detected. Increasing the tree length (sequence divergence) by a factor of three for the shorter 200 position alignments increases the number of individual splits to 350, which is still much less than observed in real data.

**Figure 5:**
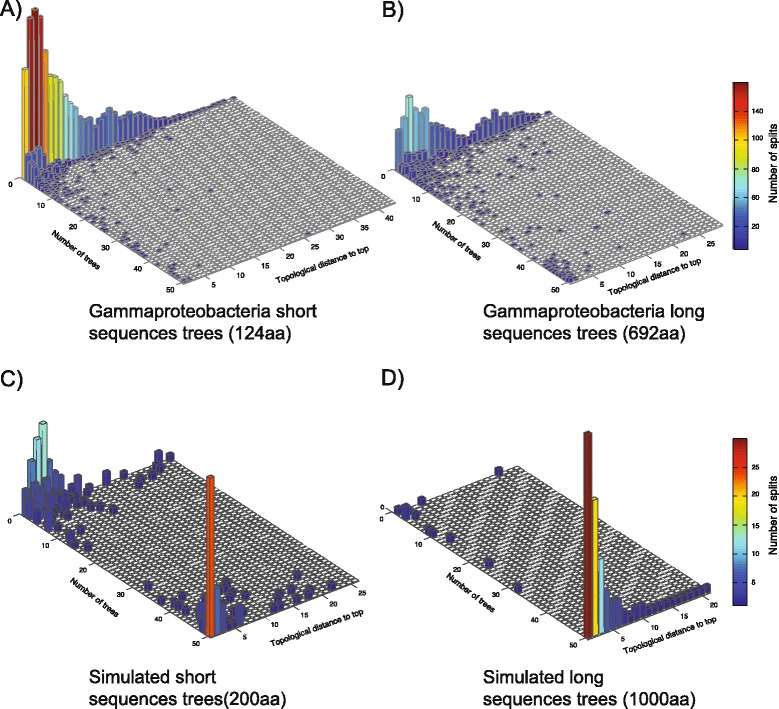
**Splits distribution in single gene data sets for real and simulated data.** For each data set all splits within single gene trees were plotted according to their topological distance to the tips of the tree and the number of trees where they are present. **A)** Dataset of 50 short gammaproteobacteria genes. **B)** Dataset of 50 long gammaproteobacteria genes. **C)** Simulated dataset with initial sequence length of 200 positions. **D)** Simulated dataset with initial sequence length of 1000 positions.

Comparing the distribution of incompatible splits between simulated data and real data make the differences more obvious (Figure 5). In real data, in this case the gammaproteobacteria, most of the observed splits appear only in one of the trees. This is true for data made from short sequences as well as for long sequence data (Figure 5A,B). Whereas in the long sequence data, the number of splits observed in single trees is strongly reduced. Within the simulated data most of the splits are present in all trees (Figure 4C,D). In the short simulated data, some splits are only present in single trees.

## Discussion

Reconstructing a single phylogenetic tree from a collection of individual genes by using concatenated alignments has long been common practice in phylogenetic analyses. Although concatenation is widely implemented, most investigations of its underlying properties are, like the present study, empirical rather than theoretical in nature [41-45]. The result is that observations and correlations can be gleaned regarding the behaviour of the data in concatenation, but the responsible causalities remain obscure.

Concatenation entails the *a priori* assumption that the individual genes in question evolved along a common phylogeny. This is often difficult to demonstrate for real data, especially for data from prokaryotes [5]. Thus, inferences that are based on concatenation trees assume — explicitly or implicitly — that the concatenated genes were not subject to processes such as recombination, gene conversion, lateral gene transfer and the like, processes that are not fundamentally tree-like in nature. Yet even when all genes follow the same phylogeny, their trees might still differ owing to variety of aspects, such as evolutionary rates, selective, structural and functional constrains, and the level of stochastic noise introduced by neutral substitutions. Such evolutionary mechanisms can lead to model misspecification even in the analysis of a single gene family. In the context of alignment concatenation, however, the problem becomes acute, since no single model can subsume all genes simultaneously, and model misspecification is more or less guaranteed. Some current methods of phylogenetic inference can deal with such factors better than others [9].

Methods for testing sets of trees for a common history is an alternative [46,47]. In our case, it remains doubtful if we could find meaningful subsets of trees. A distance matrix including all 200 gammaproteobacterial trees, sorted according to their sequence length (Additional file 9), shows that topological distance is mainly a matter of sequence length. The same is true for the fungi data (Additional file 9). So clustering algorithms might tend to cluster longer sequence trees together, due to their higher similarity. We applied a simple hierachical clustering algorithm to have an estimate what the result might look like. For each of the two datasets, the algorithm found mainly one large cluster, in which almost all trees were included. Some of the worst trees, in terms of incongruence, remain as single clusters.

The reliability of phylogenetic trees reconstructed from concatenated alignments can be assessed from two opposing perspectives. Bootstrap analysis, which originally was proposed as a methodology appropriate for single gene trees [40], can be applied to any alignment-like data, such as the concatenated alignment of several genes. This approach ignores the fact that different parts of the concatenated alignment originate from different genes, and focuses on the robustness of the estimated topology given the totality of the sequence data. An alternative approach views concatenated alignment trees as consensus-like, and focuses on the congruence between such trees and the underlying gene trees [41,42,44]. In the presence of long alignments, bootstrap analysis typically assigns high support to almost every branch of the concatenation tree while comparison to the individual gene trees indicates that congruence is observed only at the tips of the tree, and that deeper internal branches are typically highly incongruent among gene trees and between gene trees and the concatenation tree. The high bootstrap support observed here for concatenated alignments may be artificial, resulting from the large sample size and possibly biased by signals generated by a few genes. It is well-known that bootstrap and similar support values increase with the increasing number of sites sampled [41] such that a high BPs for a concatenated phylogeny does not necessarily mean that the tree is thus likely to be correct [42,44,48]. For very large data sets, phylogenetic results become increasingly dependent upon the model, rather than the number of sites sampled [9,41]. Congruence analysis, on the other hand, reveals the variety of evolutionary signals in the underlying collection of genes, and thus provides a more conservative interpretation of the phylogenomic signals, thereby informing data collation strategies.

Galtier [43] showed higher levels of congruence for eukaryotic than for prokaryotic data, similar to our present findings, and furthermore that in bacteria the congruence is slightly positively correlated to the sequence length of the chosen genes, an effect that we observed in a more pronounced manner in the present data. In a study encompassing 21 fungal species and 246 single copy genes [45], gene size was also shown to be a proxy for the phylogenetic performance of individual genes, an effect detected in all gene samples examined here. Our results also underscore the effects of sequence length on phylogenetic analysis.

Although we suspect that LGT in prokaryotes might underlie the finding that congruence between individual trees and the concatenation tree is higher for data from eukaryotic genomes than it is for prokaryotic genomes, no causal relationship can be established. We found a higher LGT rate within prokaryotic data and also an effect of this rate on congruence. But distinguishing LGT from reconstruction artefacts remains difficult, since available LGT detection programms rely on tree comparions.

## Conclusions

In general, for the prokaryotic data we observe, like others before us [15,19], a tree of tips, where the terminal branches seem well supported but the deeper branches are not recovered by any of the individual genes studied. Unexpectedly for us, this was observed recurrently for three data sets spanning very different phylogenetic depths among prokaryotes, almost in a fractal-like manner. The lack of congruence among individual genes for deeper branches, which show high BPs in the concatenated analyses, we call the "disappearing tree" effect. Its cause remains obscure, but it provides a source of many caveats when it comes to attempting to infer evolutionary events from branches with high BPs in prokaryotic genome phylogenies. If an ancient evolutionary signal is real, for example the bacteria-archaea split [49], then it should be supported by individual genes, which we observe in the present study. Concatenation is an important aspect of modern phylogenomics and is not likely to go away any time soon, it is therefore all the more important to understand the properties of concatenation and its relationship to the individual underlying trees.

## Availabilty of supporting data

The data sets supporting the results of this article are available from the Data available from the Dryad Digital Repository: doi:10.5061/dryad.06640.

## Additional files

Additional file 1:
**Taxa list for the mixed prokaryote dataset.** List of species and abbreviations used in analysis for Figure 1 a and downstream analysis. Additional file 2:
**Taxa list for the proteobacterial dataset.** List of species and abbreviations used in analysis for Figure 1 b and downstream analysis. Additional file 3:
**Taxa list for the gammaproteobacterial dataset I.** List of species and abbreviations used in analysis for Figure 1 c and downstream analysis. Additional file 4:
**Taxa list for the gammaproteobacterial dataset II.** List of species and abbreviations used in analysis for Figure 3 b and downstream analysis. Additional file 5:
**Taxa list for the fungi dataset.** List of species and abbreviations used in analysis for Figure 2 b and downstream analysis. Additional file 6:
**Taxa list for the mixed eukaryotic dataset.** List of species and abbreviations used in analysis for Figure 1 a and downstream analysis. Additional file 7:
**Correlation between alignment length, tree incongruence and bootstrap proportions. A)** Number of different splits observed in bins of 5 trees is plotted against the average alignment length. **B)** Average bootstrap proportions within one tree plotted against the average alignment length. The polynomial regression is indicated as a dotted line. Additional file 8:
**Influence of LGT rate on tree incongruence.** Number of different splits observed in bins of 5 gammaproteobacterial trees is plotted against the average alignment length. Blue dots indicate trees with an low estimated LGT rate (<= 1 event, estimated by PRUNIER ) and orange dots represent trees with an high estimated rate of LGT (>=5 event, estimated by PRUNIER ). The polynomial regression is indicated as a colored dotted line for each dataset. Additional file 9:
**Distance matrices.** Two distance matrices showing topological distances between single gene trees for fungi and gammaproteobacteria. Distances were measured by counting the number of different splits between two trees. Trees represented in the matrix are sorted according to their underlying sequence length.
