# Supplementary figures and images for: Concatenated alignments and the case of the disappearing tree
Source: BMC Evol Biol. 2014 Dec 30;14:266. doi: 10.1186/s12862-014-0266-0 (PMC4302582; doi:10.1186/s12862-014-0266-0)

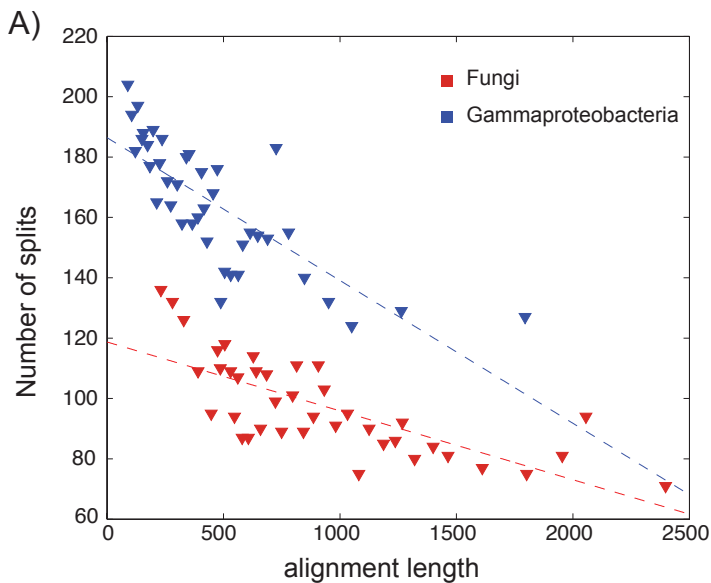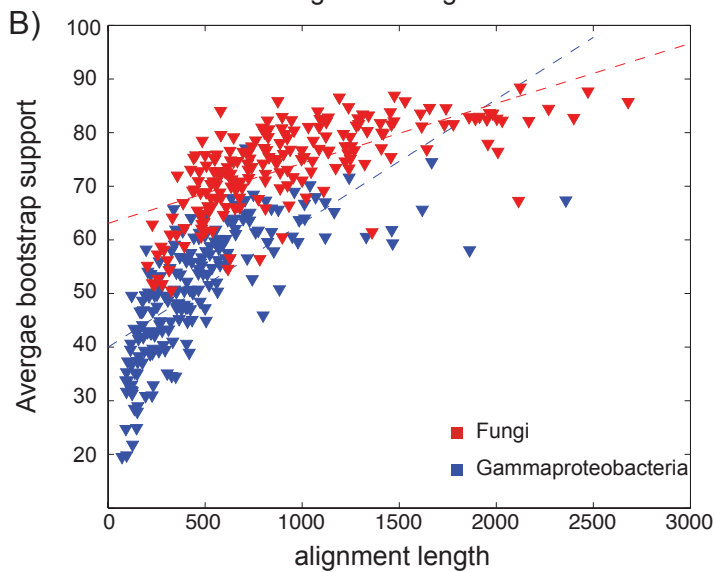

Supplement: Additional file 7: — Correlation between alignment length, tree incongruence and bootstrap proportions. A) Number of different splits observed in bins of 5 trees is plotted against the average alignment length. B) Average bootstrap proportions within one tree plotted against the average alignment length. The polynomial regression is indicated as a dotted line. [file 12862_2014_266_MOESM7_ESM.pdf]

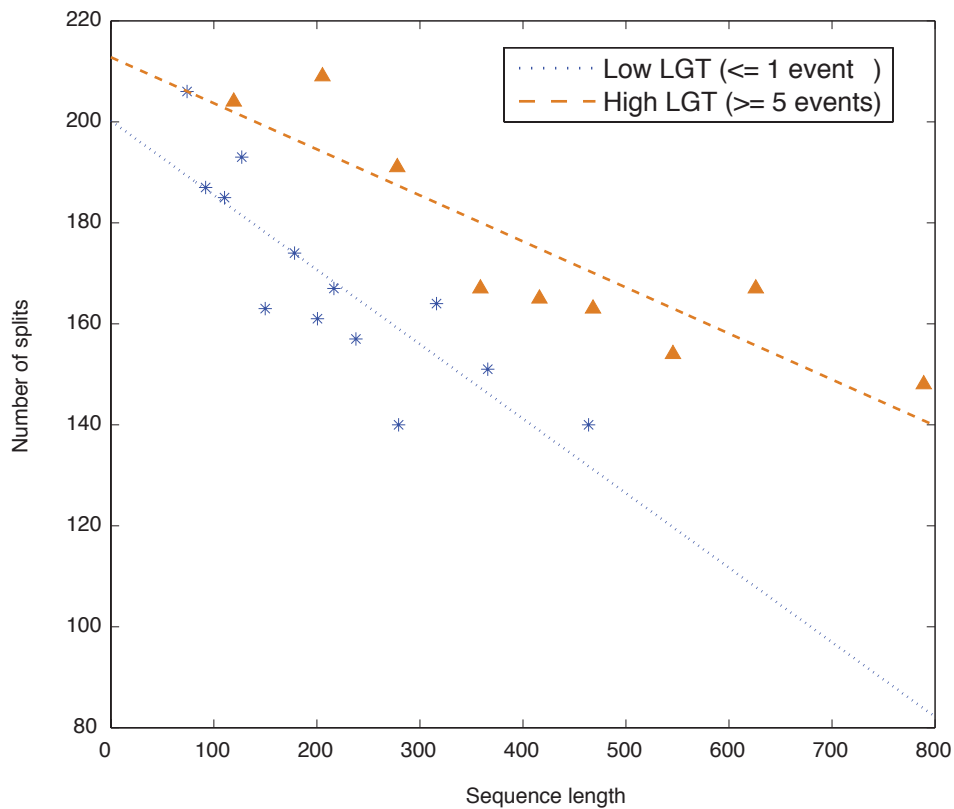

Supplement: Additional file 8: — Influence of LGT rate on tree incongruence. Number of different splits observed in bins of 5 gammaproteobacterial trees is plotted against the average alignment length. Blue dots indicate trees with an low estimated LGT rate (<= 1 event, estimated by PRUNIER ) and orange dots represent trees with an high estimated rate of LGT (>=5 event, estimated by PRUNIER ). The polynomial regression is indicated as a colored dotted line for each dataset. [file 12862_2014_266_MOESM8_ESM.pdf]

# Gammaproteobacteria

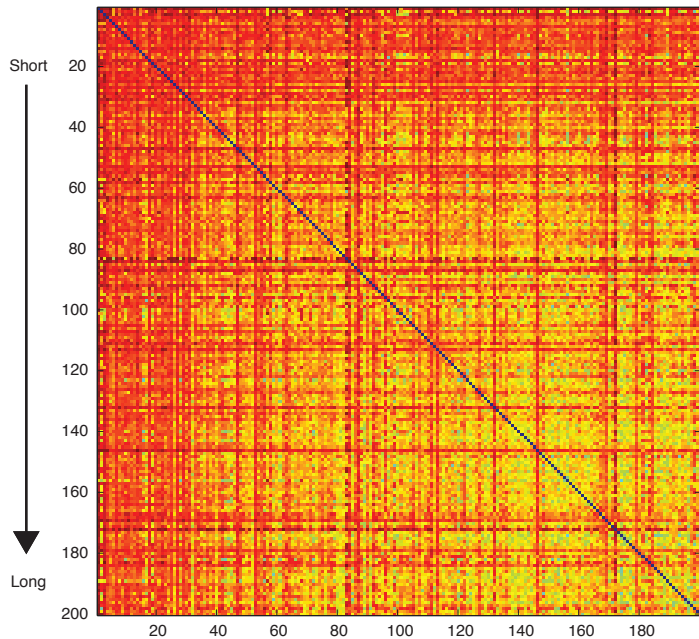

# Fungi

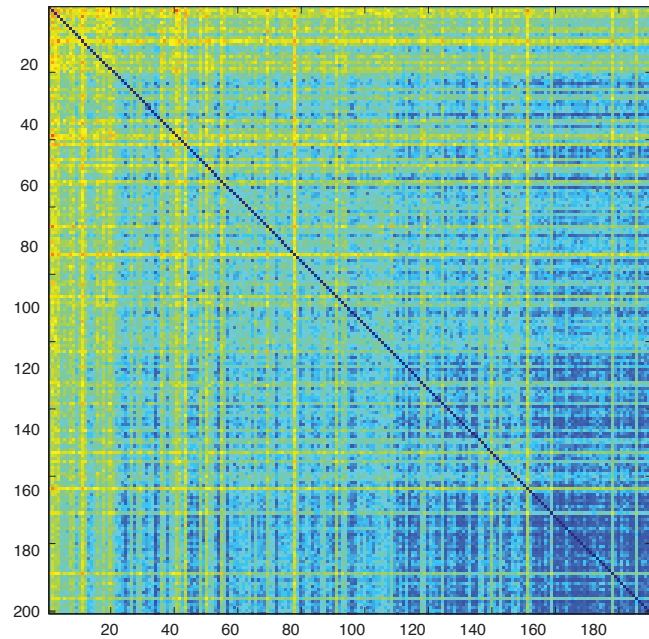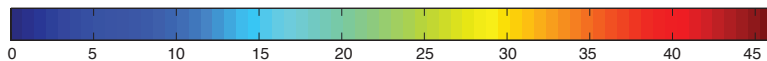

Number of different splits between trees

Supplement: Additional file 9: — Distance matrices. Two distance matrices showing topological distances between single gene trees for fungi and gammaproteobacteria. Distances were measured by counting the number of different splits between two trees. Trees represented in the matrix are sorted according to their underlying sequence length. [file 12862_2014_266_MOESM9_ESM.pdf]
